# Editorial: Exploring the future of neurology: how AI is revolutionizing diagnoses, treatments, and beyond

**DOI:** 10.3389/fneur.2025.1556510

**Published:** 2025-02-05

**Authors:** Rafeed Alkawadri, James M. Hillis, Eishi Asano

**Affiliations:** ^1^Human Brain Mapping Program, Department of Neurology, University of Pittsburgh Medical Center, Pittsburgh, PA, United States; ^2^Department of Neurology, Massachusetts General Hospital and Havard Medical School, Boston, MA, United States; ^3^Departments of Pediatrics and Neurology, Wayne State University, Children's Hospital of Michigan, Detroit, MI, United States

**Keywords:** AI, machine learning, precision medicine, accuracy paradox, SMOTE, seizure prediction, fat tail distribution, personalized care

Artificial intelligence (AI) has undergone a remarkable evolution since its conceptualization over half a century ago. Yet, despite the established theoretical foundations of machine learning, neural networks, and natural language processing, the widespread clinical application of AI in neurology, and medicine more broadly, has faced significant delays. Key barriers have included limited computational power, insufficient data quality and quantity, and a healthcare culture slow to embrace technological disruption. Recent advancements in neural architectures, the availability of large-scale datasets, and access to standardized open-source computing resources have begun to unlock AI's potential in neurology.

In this Research Topic, we explore AI's contributions to neurology, with a focus on diagnostic and therapeutic innovations and future direction. Nonetheless, AI's potential extends beyond diagnostics and treatments. It offers solutions in data science, with intelligent documentation, and enhanced patient-provider communication. For instance, large language models can already generate concise, context-rich clinical notes, with the potential to reduce clinician burnout. AI can also simplify complex medical information, fostering better patient adherence and trust.

Adopting AI in healthcare remains slow, owing perhaps to medicine's traditional nature, which often prioritizes established norms and rigorous validation among other factors. Integration into routine clinical use can take decades, requiring significant training, policy adjustments, and cultural shifts (1). As younger generations of physicians and administrators, more comfortable with digital transformation, enter the field, AI adoption is expected to accelerate (2).

The funding landscape also affects AI's integration into clinical practice. Traditional research funding often prioritizes methodological originality over iterative breakthroughs, despite the practical progress arising from refining AI tools and validating them with real-world clinical data. This mismatch may delay translation to practice (3). Partnerships with industry or venture capital could accelerate adoption, though such involvement must balance agility with adherence to regulatory and ethical standards.

AI's conceptual challenges demand careful consideration. Designing AI to mimic an “average” physician underestimates its potential to redefine medical paradigms (4). Medicine, as both an art and a science, benefits when AI transcends human limitations by identifying novel disease markers and treatment strategies (5). The optimal approach must be validated based on outcomes and fosters collaboration between physicians and AI, creating a human-machine hybrid where AI enhances human expertise with insights beyond traditional methodologies (6). The integration of AI in medicine demands robustness, reliability, and rigorous validation to ensure healthcare professionals can confidently incorporate AI insights into clinical workflows. Grounded in centuries-old principles like *primum non nocere*, medicine requires AI systems to demonstrate consistent performance across diverse populations and be evaluated using standard validation markers, similar to non-computational tools and markers. Knowledge transfer will most likely evolve alongside advancing technologies, emphasizing principles designed to account for the likely ever accelerating pace of innovation. Explainability is equally critical, enabling clinicians to interpret and challenge AI recommendations, particularly in nuanced fields like neurology. In medicine as an art, AI will more likely complement, not replace, clinical expertise, with models validated through metrics such as sensitivity, specificity, inter-model, intra-model, inter-interpreter reliability, and predictive values. Human judgment presently remains essential to capture subtleties algorithms may overlook, while oversight mitigates risks like AI confabulation and ambiguity inherent in generative or creative tasks by definition. By preserving the human touch, AI can serve as a reliable partner, not an autonomous decision-maker not the least of all by modern-day available tools or immediate tomorrow's projections.

Traditional biostatistics, which focus on hypothesis testing and p-values, are often mistakenly conflated with AI frameworks (7). AI operates on complex, multidimensional feature sets rather than simple correlations, combining weak individual features into robust predictive signals. However, emphasizing accuracy as the sole metric is problematic in medicine, where many diseases are rare. This challenge is especially true for imbalanced datasets and is known as the “accuracy paradox;” it necessitates the use of alternative metrics, such as precision, recall, F1-score, and area under the curve (AUC) for both training and validation (8, 9). Addressing class imbalance is critical to improving model performance and clinical relevance. Techniques such as oversampling minority classes (e.g., Synthetic Minority Oversampling Technique; SMOTE) or adjusting class weights can significantly enhance the robustness and applicability of AI models in clinical settings (4, 10). Temporal forecasts in neurology present additional challenges, especially as patient outcomes are influenced by multifaceted factors, including behavioral and social determinants. Time-series often exhibit “fat-tail” distributions, further complicating traditional statistical approaches (11, 12). AI can adapt to such irregularities and outliers, and emphasize adaptability and robustness. In such settings, retraining with new data can prove essential to continuously align the model with evolving patterns, particularly rare, high-impact events characteristic of fat tails. Furthermore, disease rarity need not preclude AI applications; techniques such as data augmentation, transfer learning, and federated learning can effectively model less-common cases (13).

In this collection, we aimed to provide a comprehensive update on the most recent advances in AI applications in clinical neurology. Kerr et al. presented an overview on the role of machine learning in seizure detection and forecasting, through technologies like wearable sensors, EEG-based systems, and advanced algorithms. The integration into clinical practice will necessitate addressing challenges such as false positives, deficiencies in long-term signal quality, and variability in seizure patterns. Yousefi et al. reviewed machine learning for detection and screening of neurodegenerative and neurocognitive disorders, by leveraging diverse datasets, including imaging, genetic, and clinical data. Guo et al. presented ideas for how AI could facilitate innovation with rehabilitation equipment for children with cerebral palsy by integrating into existing traditional aspects of practice. Ru et al. used data augmentation to improve seizure detection on EEG, combining Adversarial and Mixup Data Augmentation (AMDA) with a one-dimensional convolutional neural network and gated recurrent unit achieving impressive performance metrics, while Yang et al. used machine learning to predict neurological symptoms of Wilson disease employing a combination of clinical and radiologic findings and extreme gradient boosting. Fard et al. and Zhuo et al. both researched imaging applications of AI: for image synthesis of interictal SPECT from MRI and PET, and for measurement of semicircular canal spatial orientation respectively. Generative adversarial networks (GANs) were used to synthesize interictal SPECT images from MRI and PET, achieving high-quality results and demonstrating that MRI can produce reliable SPECT images, potentially eliminating the need for additional scans and reducing patient exposure to radiation. Xu et al., investigated the identification of middle cerebral artery stenosis using transcranial Doppler, with mixed performance metrics and high sensitivity, while Wen et al. predicted early neurological deterioration after intravenous thrombolysis, with models highlighting “Onset to Needle Time” and “Admission NIHSS Score” as predictors, showcasing their potential to enhance individualized stroke care and refine post-thrombolysis risk stratification. A common theme reflected in the included articles was that the integration of these models and technologies into clinical practice requires rigorous validation, addressing challenges like data variability, and interpretability to ensure scalable and equitable benefits in early diagnosis and disease management.

In conclusion, AI's transformative potential in neurology extends well beyond diagnostics. By harnessing its capabilities in data stewardship, collaborative frameworks, and predictive modeling, while addressing cultural, funding, and methodological challenges, we can create a more dynamic and adaptive neurological practice. At this critical juncture, business and venture models are emerging as equally important as traditional academic funding in driving progress. Ultimately, the integration of AI alongside physicians' clinical expertise promises to usher in a new era of neurology, defined by greater precision and more personalized care.
